# Effects of lanthanum and acid rain stress on the bio-sequestration of lanthanum in phytoliths in germinated rice seeds

**DOI:** 10.1371/journal.pone.0197365

**Published:** 2018-05-15

**Authors:** Yong Si, Lihong Wang, Qing Zhou, Xiaohua Huang

**Affiliations:** 1 State Key Laboratory of Food Science and Technology, School of Environment and Civil Engineering, Jiangsu Key Laboratory of Anaerobic Biotechnology, Jiangnan University, Wuxi, Jiangsu Province, China; 2 Jiangsu Cooperative Innovation Center of Water Treatment Technology and Materials, Suzhou University of Science and Technology, Suzhou, Jiangsu Province, China; 3 Jiangsu Collaborative Innovation Center of Biomedical Functional Materials, Jiangsu Key Laboratory of Biomedical Materials, School of Chemistry and Materials Science, Nanjing Normal University, Nanjing, Jiangsu Province, China; Hainan University, CHINA

## Abstract

REEs in the environment can be absorbed by plants and sequestered by plant phytoliths. Acid rain can directly or indirectly affect plant physiological functions. Currently, the effects of REEs and acid rain on phytolith-REEs complex in plants are not yet fully understood. In this study, a high-silicon accumulation crop, rice (*Oryza sativa* L.), was selected as a representative of plants, and orthogonal experiments were conducted under various levels of lanthanum [La(III)] and pH. The results showed that various La(III) concentrations could significantly improve the efficiency and sequestration of phytolith La(III) in germinated rice seeds. A pH of 4.5 promoted phytolith La(III) sequestration, while a pH of 3.5 inhibited sequestration. Compared with the single treatment with La(III), the combination of La(III) and acid rain inhibited the efficiency and sequestration of phytolith La(III). Correlation analysis showed that the efficiency of phytolith La(III) sequestration had no correlation with the production of phytolith but was closely correlated with the sequestration of phytolith La(III) and the physiological changes of germinated rice seeds. Phytolith morphology was an important factor affecting phytolith La(III) sequestration in germinated rice seeds, and the effect of tubes on sequestration was more significant than that of dumbbells. This study demonstrated that the formation of the phytolith and La(III) complex could be affected by exogenous La(III) and acid rain in germinated rice seeds.

## Introduction

Rare earth elements (REEs) are widely used in modern industry and agriculture [1–3]. The long-term exploitation and broad use of REEs have triggered intensive geologic prospecting for rare earth ore deposits. However, the growing release of REEs in the environment may jeopardize various biotic systems [4–6]. Currently, in many countries and areas around the world (such as China, Australia and Japan), the average REE concentration in the soil ranges from 97.57 mg/kg to 322.5 mg /kg [7–12]. Compared with other REEs, the concentrations of lanthanum [La(III)] in the environment and micro-fertilizers are higher [13], so most toxicological studies have focused on the uptake of La(III) by plants. Physiological studies show that La(III) has a *hormesis* effect on plant physiological function [14]. Acid rain is also an important environmental issue [15–18]. The pH of acid rain in many countries and areas ranges from 4.2 to 4.5 [19, 20]. The average pH of acid rain ranges from 3.0 to 4.5 in China [21]. Soil is the final destination of acid rain, and studies show that acid rain is an important factor influencing the lower pH values of soil [22]. Acid rain exerts deleterious effects on most plants [23, 24] and affects the distribution of plants [25, 26]. In the southern area of the Yellow River in China, persistent acid rain pollution has caused forest degradation [27]. Our previous studies have proven that mild acid rain (pH 4.5) inhibits rice seed germination [28], while the combination of severe acid rain (pH < 3.5) and REEs aggravates the toxic effects of these elements on germinated rice seeds [29].

Phytoliths, which are also referred to as opals, consist of hydrated amorphous silica that precipitates in or between the cells of living plants via transpiration [30–32]. Phytoliths are extremely small (particle size ranging from 2 to 2000 μm) and exhibit diverse morphotypes [33, 34]. Phytoliths can encapsulate living organelles of plants, such as chloroplasts, mitochondria, plasmids, and other organelles [35]. The organelles encapsulated by plant phytolith are easily contaminated by lanthanum [La(III)], and phytolith absorbs La(III) on its surface or sequesters it inside [30]. Environmental pH is also an important factor influencing phytolith and can affect the solubility and surface charge of amorphous silica [36–38]. The solubility and stability of various phytolith morphologies are different [39]. All of these factors may affect a series of functions in plants, such as material transport, energy exchange and information transmission. Rice (*Oryza sativa* L.) is a typical high-silicon (Si) accumulation crop [40–43]. Compared with other rice organs, the quantity of phytolith in rice’s husk is the largest [40, 44]. After crushing five genotypes of rice grains (including husked and milled rice), Li et al. [45] found that the elemental contents of C, Al, Mg and Fe in phytolith are 2.11%~2.89%, 0.011%~0.022%, 0.005%~0.008% and 0.005%~0.018%, respectively. At present, the relationship between exogenous La(III), pH, phytolith morphology and phytolith REE sequestration remains unclear, and studies on REE sequestration by phytoliths in rice grains have not yet been reported.

The seed germination stage is a crucial life period of plants [46] and determines the yield and quality of mature plants [47–49]. In this study, germinated rice seeds were selected as the research object, and La(III) was selected as the representative REE. Using a series of experimental methods such as the microwave digestion method and inductively coupled plasma atomic emission spectrometry (ICP-AES), this study revealed the effects of La(III) and pH on phytolith La(III) sequestration, phytolith morphology and the germination physiological index of germinated rice seeds and discussed the reasons for La(III) sequestration by phytolith, thus providing references for further understanding the mechanisms of plant phytolith La(III) sequestration and objectively evaluating the environmental risk of rare earth pollution.

## Materials and methods

### Culture methods for plant materials

The rice seeds (a hybrid japonica rice, cv. Huai-8) were disinfected with 3% hydrogen peroxide for 30 min and then washed with deionized water several times. The seeds were then soaked in deionized water and placed in a constant-temperature incubator at 25°C for 24 h before being washed again with deionized water. According to the actual concentration of La(III) in the current environment [3, 10, 11], along with the rice soil pH used at the International Rice Research Institute [50, 51] and the actual pH value of acid rain in the environment [21], we employed four different LaCl_3_ concentrations (0, 20, 100 and 300 mg/L) and three pH values (5.5, 4.5 and 3.5) to simulate the combined stress of bio-available La [LaCl_3_, La(III)] and acid rain. In this study, a La(III) concentration of 20 mg/L represented the low La(III) concentration, while a La(III) concentration of 100 or 300 mg/L represented the high La(III) concentration. Acid rain stock solutions were prepared using a solution of concentrated H_2_SO_4_ and HNO_3_ at a ratio of 3:1 (v/v, by chemical equivalents) in accordance with the general anion composition of rainfall in China (2007–2008) [52]. Therefore, we developed 12 treatments, and each treatment included four culture dishes representing four biological repeats (see Table 1). Two layers of evenly laid filter paper were placed in the bottom of each culture dish, upon which 50 rice seeds were placed. A total of 2400 rice seeds (4×12×50) were used in this study. A 3/4-strength culture solution was then added to the dishes, which were placed in a constant-temperature incubator. The incubator was maintained at a temperature of 35°C during the daytime for 13 h and at 25°C during the nighttime for 11 h. The seeds were allowed to germinate for 7 d.

**Table 1:** Various La and pH treatments in the experimental design.

Treatments	pH	LaCl_3_ (mg/L)	Treatments	pH	LaCl_3_ (mg/L)
T1 (CK)^a^	5.5	0	T7	4.5	100
T2	5.5	20	T8	4.5	300
T3	5.5	100	T9	3.5	0
T4	5.5	300	T10	3.5	20
T5	4.5	0	T11	3.5	100
T6	4.5	20	T12	3.5	300

^a^ T1 (CK) means control.

### Determination of phytolith content and morphology

The rice seeds were germinated under stress for 7 d. Thereafter, the seeds were rinsed in an ultrasonic bath for 15 min, followed by rinsing with ultrapure water. The seeds were then dried at 105°C for 20 min, followed by 75°C until constant weight. All of the seeds were milled in preparation for measurements.

A microwave digestion method was used to extract phytoliths from plant samples according to Parr et al. [53] with Walkley-Black-type digestion [54]. Briefly, 0.2 g of milled seeds was microwave digested with 3 mL HNO_3_, 2 mL H_2_O_2_ and 0.5 mL HCl at 90 °C for 3 min. The sample was then centrifuged, and the supernatant was decanted. The centrifugation-decanting steps were repeated three times with double-deionized water. The solid residue (extracted phytoliths) remaining was dried to a constant weight in a drying oven at 75°C, sealed in a pre-weighed centrifuge tube and weighed to calculate the phytolith content. The method for calculating phytolith content is shown in formula 1 and is presented in the literature [30, 55]. To observe phytolith morphology, we used the heating digestion method described by Lu et al. [56]. Briefly, 0.5 g of milled seeds was placed in 20 mL of saturated nitric acid for over 12 h. The centrifugation steps were identical to the microwave digestion method above. Uniform liquid samples of 5 μL obtained from plant digestion were mounted onto microscopic slides with Canada Balsam medium for photomicrography and in liquid medium for counting and measuring. Light photomicrography at 400× magnification was used to determine the silicon structure patterns on each slide [57]. Every phytolith morphotype was recorded in 30 randomly selected grains in each uncovered culture dish. The content of phytolith morphology in germinated seeds was calculated using formula 2.

$$\text{Phytolith}\;\text{content}\;(\%)=\frac{\text{Weight}\;\text{of}\;\text{phytoliths}\;(\text{g})}{\text{Dry}\;\text{weight}\;\text{of}\;\text{rice}\;\text{seeds}\;(\text{g})}\times 100$$ (1)

$$\text{Content}\;\text{of}\;\text{phytolith}\;\text{morphology}\;(\%)=\frac{\text{Count}\;\text{of}\;\text{one}\;\text{phytolith}\;\text{morphotype}}{\text{Count}\;\text{of}\;\text{all}\;\text{phytolith}\;\text{morphotypes}}\times 100$$ (2)

### Determination of PhytLa content

Phytoliths and plant samples were extracted with a modified lithium metaborate fusion method [58, 59]. The samples were placed in a high-purity graphite crucible, stirred homogeneously with lithium metaborate, and then combusted in a muffle furnace. During the ashing process, the temperature was gradually increased to 200°C and sustained for 10 min, then further increased to 500°C and sustained for 5 min, and finally heated to 950°C and sustained for 15 min [30]. After cooling, the ash was dissolved in dilute nitric acid, and dissolution was accelerated using a magnetic stirrer. The phytolith-sequestered La (PhytLa) were determined via inductively coupled plasma-atomic emission spectroscopy (ICP-AES). All measurements mentioned above were monitored with controls consisting of a standard soil sample (GBW07405, GSS-5) and a standard plant sample (GBW 07603, GSV-2). A precision of better than 5% was achieved through analysis of the standard samples. Here, the PhytLa content represents the percentage of La in phytoliths by weight (%, wt). The PhytLa content of seeds (PLCS, %, wt) was calculated with formula 3. The contribution rate of phytolith to the PhytLa content (PPLCR, wt) was calculated by formula 4, the larger the PPLCR value, the greater the contribution of phytolith to phytolith La(III) sequestration, and vice versa.

$$\text{PLCS}\;(\%)=\frac{\text{Phytolith}\;\text{content}\times \text{PhytLa}\;\text{content}}{\text{100}}$$ (3)

$$\text{PPLCR}=\frac{\text{PhytLa}\;\text{content}}{\text{Phytolith}\;\text{content}}$$ (4)

### Analytical method for seed vigor

The number of germinated seeds was counted daily in the laboratory for 7 d. Germinal length (GL, mm), root length (RL, mm), fresh weight (FW, g) and dry weight (DW, g) were measured after the 7 d germination period. Seed samples were washed with deionized water and then dried at 80°C to a constant weight to determine DW. Other indicators of seed germination were calculated using the formulas presented in Table 2 [60–62].

**Table 2:** Formulas for the calculation of indicators of seed germination.

No.	Indicators	Abbreviations	Calculation formulas
5	*Germination rate (%)*	GR	(Number of germinated seeds for 7d÷Total number of seeds)×100
6	*Germination energy (%)*	GE	(Number of germinated seeds for 3d÷Total number of seeds)×100
7	*Germination index*	GI	Σ (Gt÷t)
8	*Abnormal germination rate (%)*	AGR	(Number of abnormal germinated seeds÷Total number of seeds)×100
9	*Storage material loss rate (%)*	SMLR	(Seed weight before germination-total dry weight of all parts of germinated seeds)÷Seed weight before germination×100
10	*Storage material operation efficiency (%)*	SMOE	Seed dry weight (shoots+roots)÷Dry weight of all seed parts (shoots+roots+grains)×100
11	*Germinal length inhibition index (%)*	GII	(Control germinal length-Treatment germinal length)÷Control germinal length×100
12	*Root length inhibition index (%)*	RII	(Control root length-Treatment root length)÷Control root length×100

Here, GR (see formula 5) is the percentage of the number of seeds that germinated normally within 7 d among the total number of seeds in each treatment. GE (see formula 6) is the percentage of the number of seeds that germinated normally within 3 d among the total number of seeds in each treatment. With respect to formula 7, GI is a modified Timson’s index of germination velocity, where Gt is the percentage of seed germination at 1 d intervals, and t is the total germination period [62]. The greater the value, the more rapid the seed germination. AGR in formula 8 refers to the percentage of the number of seeds that germinated containing only a shoot, and no roots, after 7 d among the total number of seeds in each treatment. With respect to GII and RII in formulas 11 and 12, the shoot length (GL) and RL of each seed should be measured, and the average length of the shoot or root in each dish should be calculated.

### Statistical methods

All data were analyzed via one-way ANOVA, and significant differences were checked using Duncan’s multiple range test. Excel and SPSS software were employed in the statistical analysis of all data and mapping.

## Results and discussion

### Relationship between phytolith formation and phytolith La(III) sequestration in germinated rice seeds

**Fig 1:**
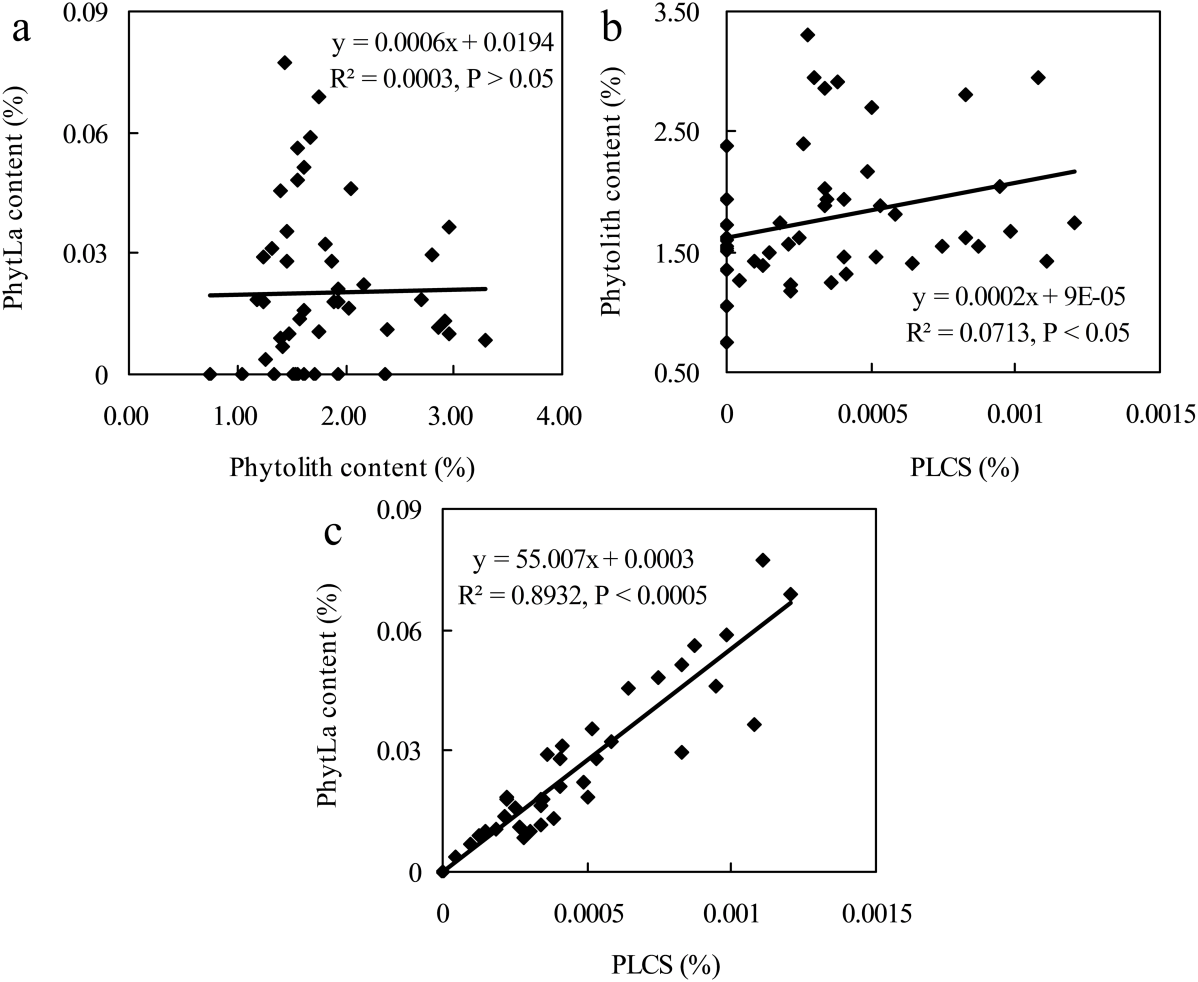
Correlations among PhytLa content, phytolith content and the PhytLa content in seeds. a PhytLa content vs. phytolith content; b phytolith content vs. the PhytLa content in seeds; c PhytLa content vs. the PhytLa content in seeds. n = 48.

The key to understanding the effects of REEs on plants is to determine the sequestration mode of REEs in plants, that is, in what form does REEs exist in plants? Kamenik et al. [63] found that phytolith extracted from plants contains REEs. In previous studies, we have proposed a possible mechanism of La(III) sequestration by phytolith [30] by which La(III) can be sequestered by phytolith through the adsorption of ions or encapsulation of organelles. However, the relationship between phytolith formation and phytolith La(III) sequestration in germinated rice seeds remains unclear, and related reports have not been found. In this study, Fig 1a shows that the correlations between the PhytLa content and phytolith content were not clear (R^2^ = 0.0003, P > 0.05). These correlations indicated that the variation in the content of PhytLa could not be attributed to the variation in phytolith content during rice seed germination, meaning that although there is a larger quantity of phytolith in rice grains [45], this quantity cannot determine the efficiency of La(III)-sequestration by phytolith. Carbon is an important element in phytoliths. However, in many previous studies, PhytC is not related to the Si content in many crops, such as sugarcane (*Saccharum officinarum* L.), bamboo (*Phyllostachys bambusoides* f. spectabilis), wheat (*Triticum aestivum* L.), and millet (*Panicum miliaceum* L.) [64–67]. Considering the close relationship between Si content and phytolith content [55, 68], this relation is in accordance with the above results, suggesting that the sequestration mechanism of PhytC is similar to that of PhytLa. Specifically, our correlation analysis revealed that the PLCS exhibited a correlation with the content of phytoliths (R^2^ = 0.0713, P < 0.05), and the PLCS was highly and significantly correlated with the content of PhytLa (R^2^ = 0.8932, P < 0.0005) (Fig 1b and 1c). These results were similar to previous findings on PhytC [45, 69, 70], which means that the final production of phytolith La(III) sequestration is determined not only by the efficiency of La(III) sequestration by phytoliths but also by the actual production of phytoliths during rice seed germination.

### Effects of the quantity and size of phytolith morphology on phytolith La(III) sequestration in germinated rice seeds

**Fig 2:**
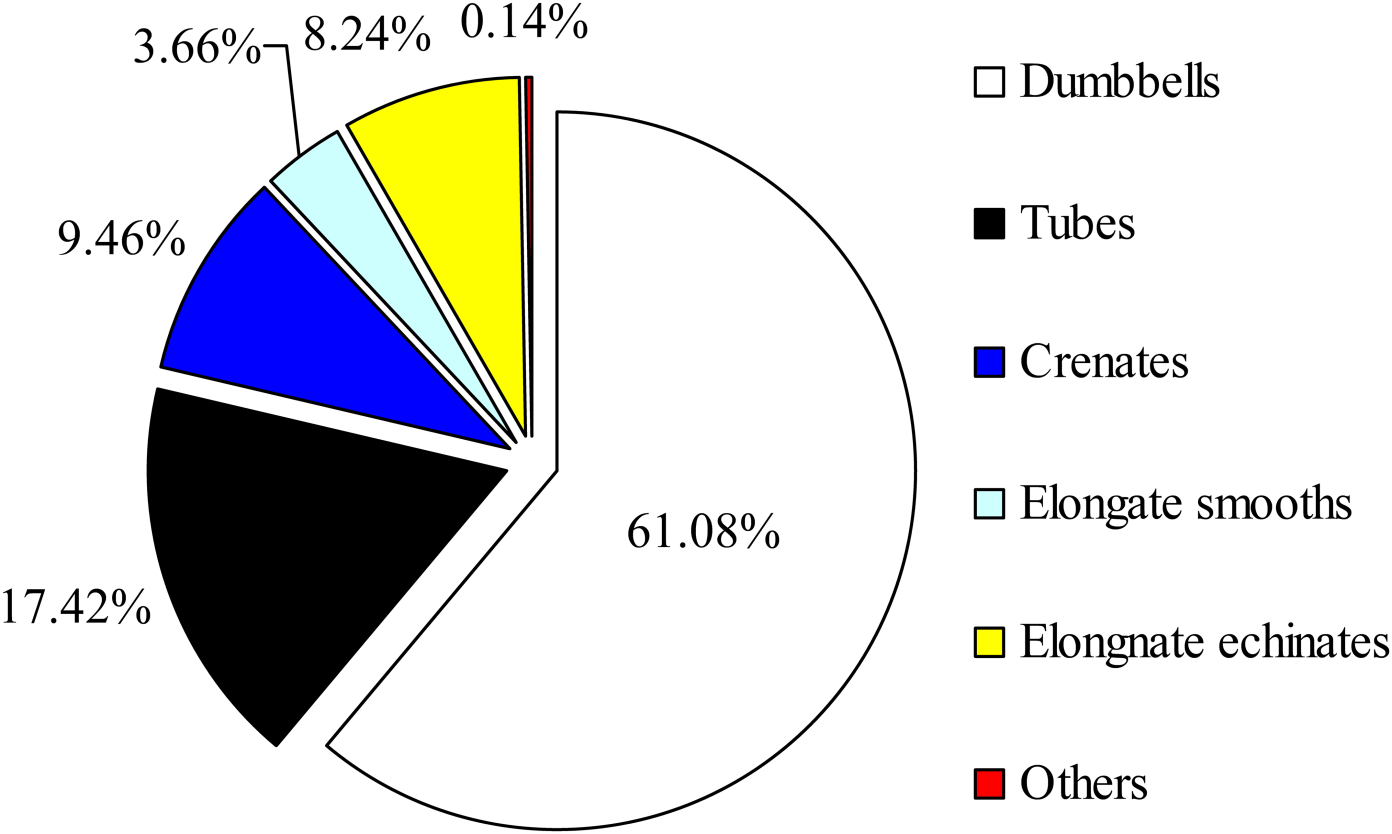
The pie chart of the quantitative proportion of different phytolith morphologies. The total number of phytolith morphotypes is 7,488.

**Fig 3:**
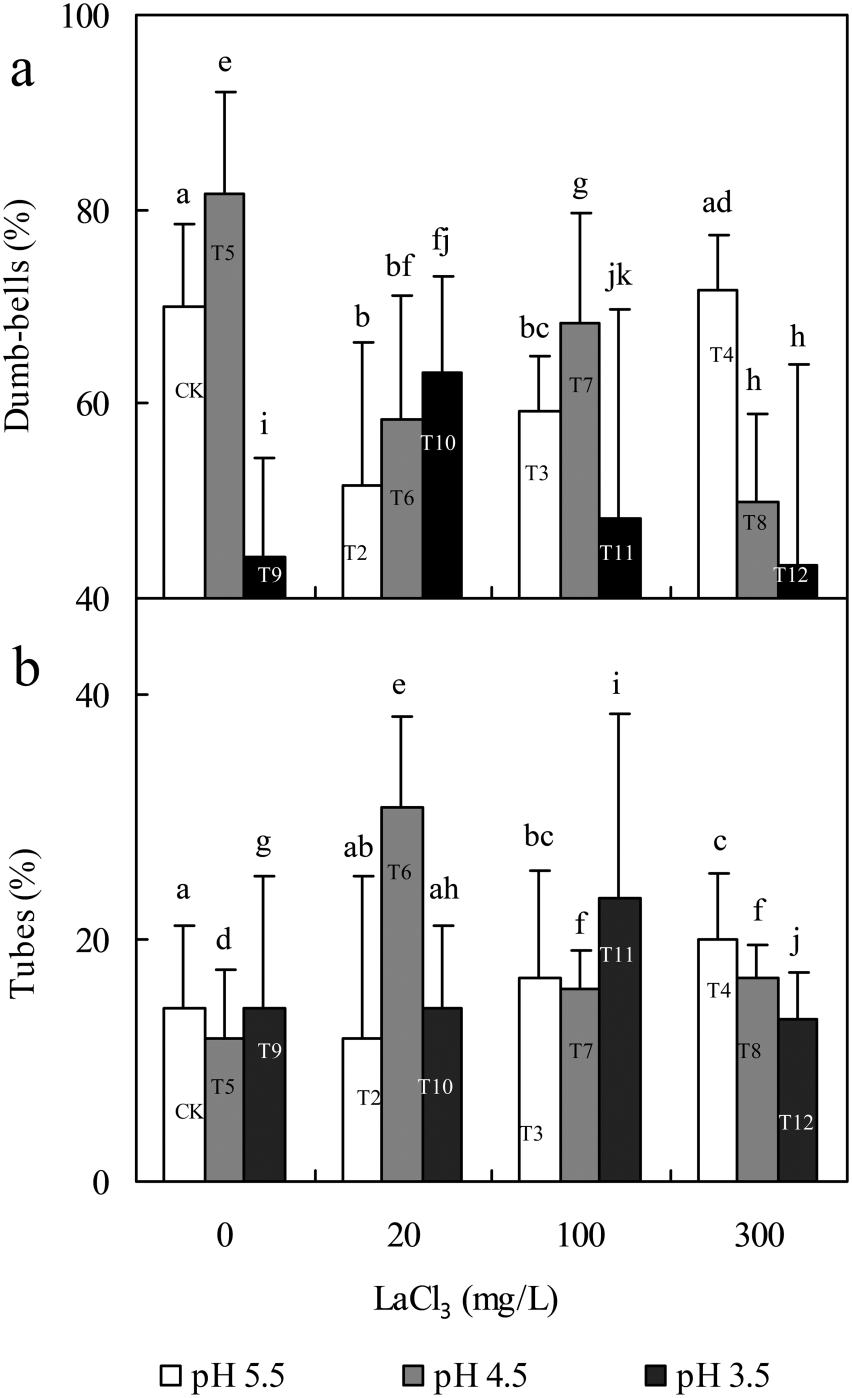
Effects of La (at a LaCl_3_ concentration of 0, 20, 100 or 300 mg/L) and acid rain (at a pH of 3.5, 4.5 or 5.5) on dumbbell (a) and tube (b) in germinated rice seeds. Error bars are standard deviations (n = 4); different letters indicate significant differences between different treatments at P = 0.05 based on the least significant difference (LSD) test.

**Fig 4:**
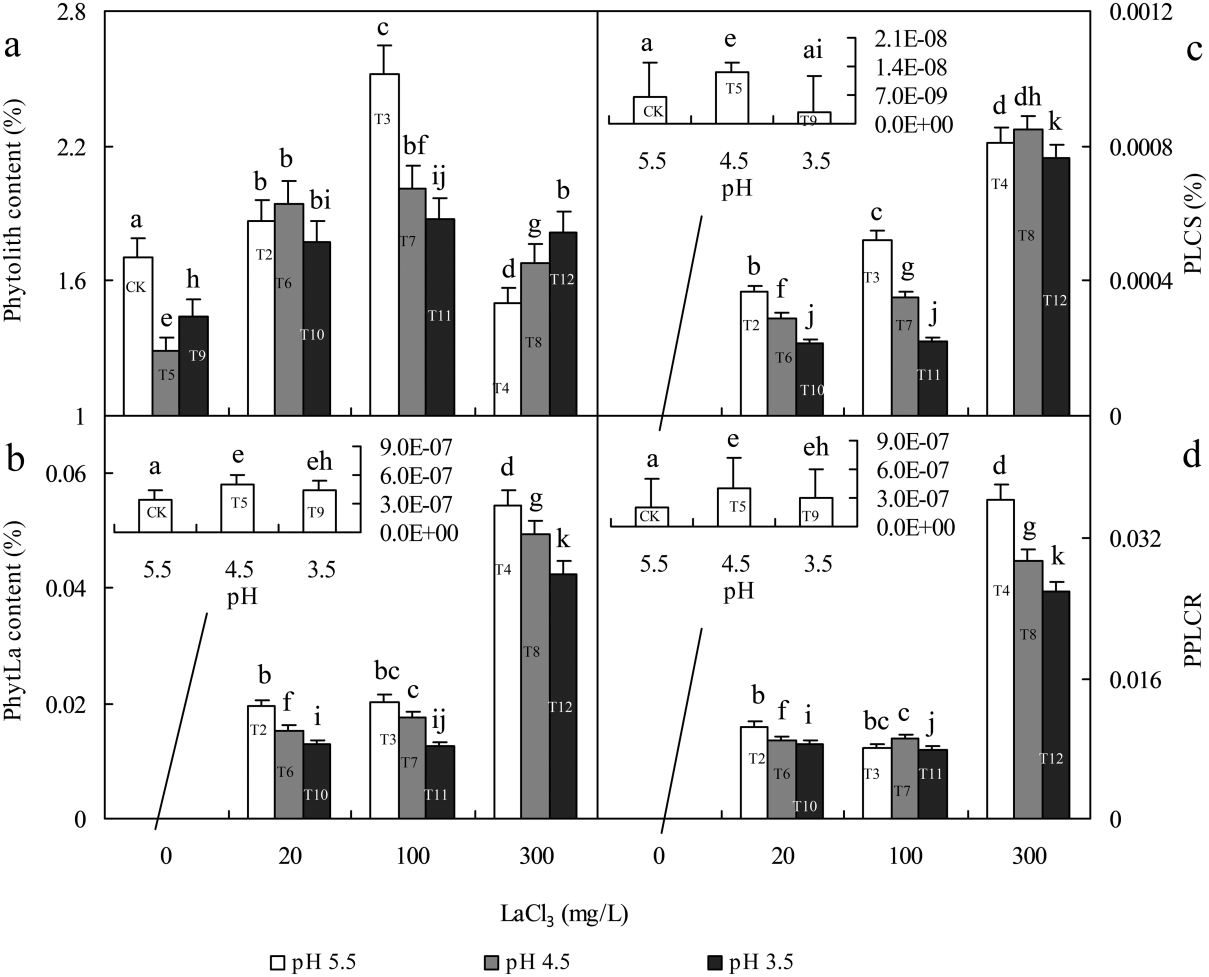
Effects of La (at a LaCl_3_ concentration of 0, 20, 100 or 300 mg/L) and acid rain (at pH value of 3.5, 4.5 or 5.5) on phytolith content (a), PhytLa content (b), the PhytLa content in seeds (c) and the PhytLa content/phytolith content ratio (d). Error bars are standard deviations (n = 4); different letters indicate significant differences between different treatments at P = 0.05 based on the least significant difference (LSD) test.

The morphology of phytolith in plants is very important because it is closely related to the epidermal structure of plants [71] and is an important factor influencing the sequestration and binding effects of phytolith on its inclusions [30]. Rice hulls are highly silicified polymers of dead cells [72] and constitute the main deposition site of Si (including phytoliths) [73]. Due to the diversity of plant cell morphologies, phytoliths exist as various morphotypes. Short cells in the epidermis of graminaceous plants (such as bamboo and rice) can be preferentially silicified [74, 75]. In the present study, dumbbells derived from the short cells of rice seeds accounted for the majority of the total phytoliths (61.08%), which confirms the above opinion (Fig 2). However, dumbbells were not the main contributor to phytolith La sequestration, while tubes with the second largest number (17.42%) were. Comparing the content of dumbbells and tubes (Fig 3) with the phytolith La(III) sequestration data (Fig 4), we found the following evidence:

First, in the single treatment with acid rain (T5 or T9), compared with CK, the contents of dumbbells, PhytLa, PLCS and PPLCR in T5 all increased significantly, but the content of tubes decreased. Compared with T5, the content of dumbbells, the PhytLa content, PLCS and PPLCR in T9 all decreased significantly, but the content of tubes increased, proving that acid rain promoted the effect of dumbbells on phytolith La(III) sequestration but inhibited that of tubes. However, the effect was different in other treatments. Second, in the single treatment with a low concentration of La(III) (T2), the content of dumbbells decreased compared with the CK, but the content of tubes did not change, and the contents of phytoliths and PhytLa and the PLCS and PPLCR increased. In the combined treatment with low La(III) and acid rain (T6 and T10), the content of dumbbells decreased compared with the CK; however, the content of tubes increased, and the contents of phytoliths and PhytLa and the PLCS and PPLCR also increased. Third, in the single treatment with 100 mg/L La(III) (T3), the content of dumbbells decreased compared with the CK, whereas the content of tubes increased and the contents of phytoliths and PhytLa and the PLCS and PPLCR increased. In the combined treatment with 100 mg/L La(III) and acid rain (T7 and T11), the content of dumbbells decreased compared with the CK; however, the content of tubes increased, and the contents of phytoliths and PhytLa and the PLCS and PPLCR also increased. Fourth, in the single treatment with 300 mg/L La(III) (T4), the content of dumbbells did not change compared with the CK, while the content of tubes increased more, and the contents of PhytLa and PLCS and PPLCR also increased, but the content of phytoliths decreased. In the combined treatment with 300 mg/L La(III) and acid rain (T8 and T12), the content of dumbbells decreased compared with the CK, whereas the content of tubes increased, and the contents of phytoliths and PhytLa and the PLCS and the PPLCR increased, the opposite variation tendency of the content of dumbbells and tubes was clear. In summary, these results indicated that with the influence of a single La(III) treatment or the combined influence of La(III) and acid rain, the number of tubes was more closely related to the efficiency and degree of phytolith La(III) sequestration, but the number of dumbbells was not. This result was similar to those of our previous study on the effects of La(III) and Si stress on rice seedlings [30]. The reason for this similarity may be related to the important transport function of vessel cells. We infer that vessel cells are exposed to more free-state La(III) due to the transport of water and elements in plants, which results in the promotion of the efficiency of La(III) sequestration by phytoliths deposited around the tubular structures. In addition, under the influence of the single acid rain treatment, the contribution of dumbbells was greater than that of tubes. The reason for this result might be related to the different formation processes; dumbbells are formed from mild-silicified cells in rice tissues [76], whereas tubes are formed from highly silicified cells. Ma [77] suggests that silicified cells (dumbbells) in rice might be partially transformed into larger silicon bodies (such as tubes). Dumbbells might respond to pH more sensitively than tubes. Therefore, the different formation processes might be among the vital reasons for the different contributions of various phytolith morphologies to phytolith La(III) sequestration.

**Fig 5:**
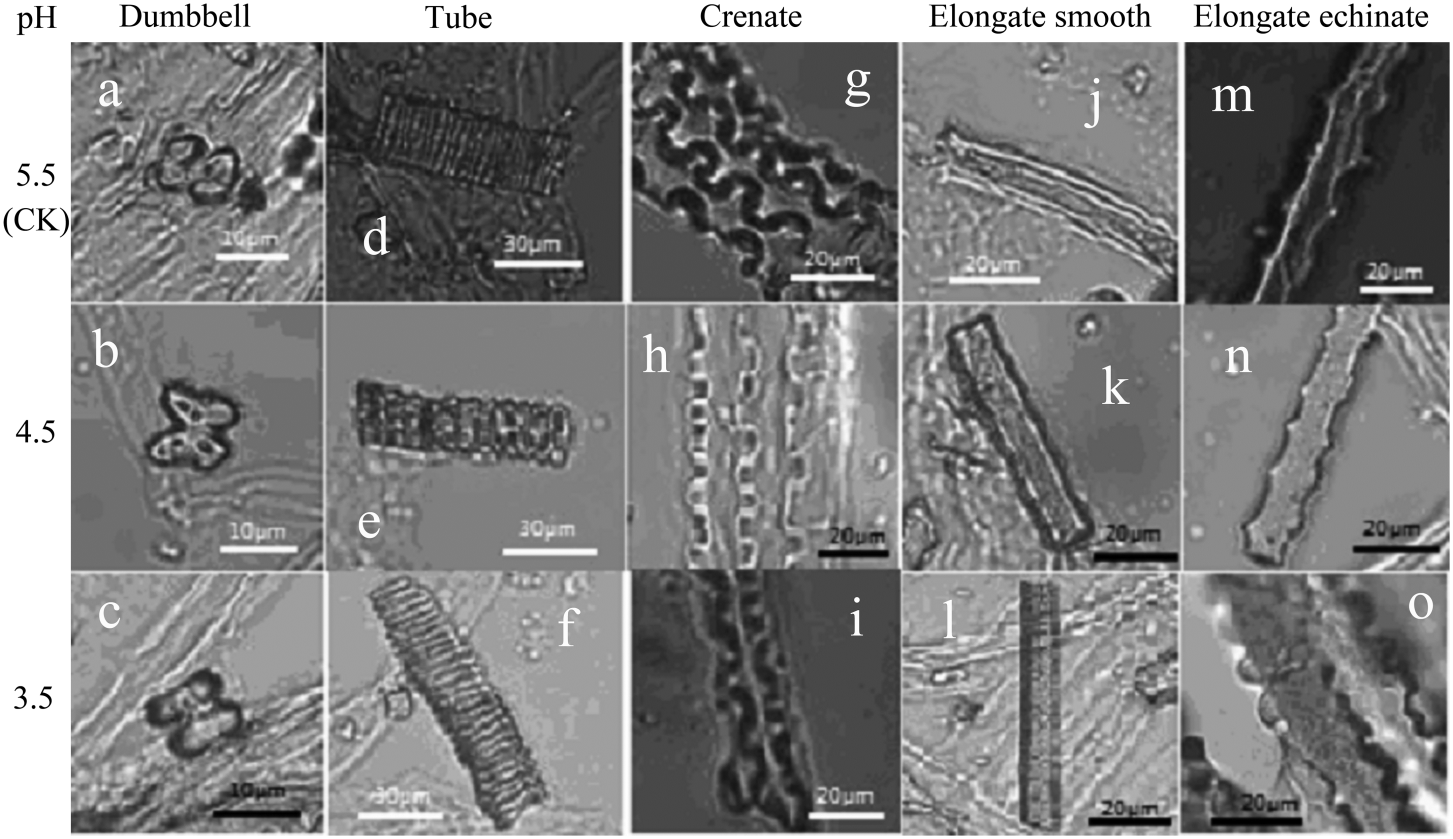
Effects of different pH levels on phytolith morphology (here, the LaCl_3_ concentration was 0 mg/L). The pH values from top to bottom are 5.5, 4.5 and 3.5, and the phytolith morphotypes from left to right are dumbbells, tubes, crenates, elongate smooths and elongate echinates.

Phytolith morphology is closely related to the position and shape of plant cells [78]. According to the variations of different phytolith morphologies under various pH levels in this study (Fig 5), we derived several conclusions. First, in the single treatment with acid rain (pH 3.5 or 4.5), dumbbells were flatter and wider (Fig 5b and 5c) compared with the CK (Fig 5a). We deduced that acid rain (pH 3.5 or 4.5) decreased the epidermal cell activity of rice seeds and slowed cell division, which caused epidermal cells to become larger and wider; hence, larger dumbbells were generated. Although many dumbbells as well as both long and short cells in the epidermis and hulls of rice seeds were observed, due to the reduction of cell functional activities, the abundance of dumbbells did not make a greater contribution to La(III) sequestration. Similar reasoning applies to the elongate echinates (Fig 5a, 5n and 5o). Second, in the single treatment with acid rain (pH 3.5 or 4.5), tubes, crenates and elongate smooth phytoliths were narrower and smaller (Fig 5e, 5f, 5h, 5i, 5k and 5l) compared with the CK (Fig 5d, 5g and 5j). The reason might be that acid rain promotes the physiological metabolism of rice seeds and causes the template cells of phytoliths (such as vessel cells, bulliform cells and tufted cells) to perform more functional tasks [79]. This increased performance results in accelerated cell division, generating both smaller single cells and smaller phytoliths. The increase in the functional tasks of cells causes more phytoliths (such as tubes and crenates) to be involved in La(III) sequestration. Therefore, acid rain (pH 3.5 or 4.5) can affect La(III) sequestration by changing the size of phytoliths, and morphological variation in phytoliths is an important factor affecting La(III) sequestration during rice seed germination.

### Effects of La(III) and acid rain on La(III) sequestration by phytolith in germinated rice seeds

Phytolith La(III) sequestration is essential for plant growth and development. However, the effects of exogenous La(III) and acid rain on phytolith La(III) sequestration in plants remain unclear, yet no report on the relationship between phytolith La(III) sequestration and the physiological changes in germinated rice seeds has been found. In this study, to be concise, we selected 4 representative germination indexes (GI, RL, FW and DW) of rice growth to reflect the physiological changes in germinated rice seeds. GI represents the Timson index, which affects the probability and growth rate of the germination of rice seeds [62]; the higher the index is, the higher the probability of seed germination and the faster growth rate are, and vice versa. As seed embryo roots can first break through the seed coat and grow in the soil and develop into roots, they are the basis for root formation. Silicon absorption in rice is realized by silicon transporter protein in roots [80, 81] and is mainly active absorption, so RL can reflect the silicon absorption ability in the later growth stages of rice. FW represents the change in the energy consumption of the physiological activities in rice seeds; the more energy consumption there is, the smaller the FW is, and vice versa. DW represents the material accumulation in rice seeds, and the variation in material accumulation can affect phytolith formation in rice seeds. In Table 3, except for DW, there were significant correlations among the 11 germination indexes, showing that the other 8 indexes could also reflect the physiological changes in the germinated rice seeds in addition to the 4 selected indexes above (other indicator data can be found in Supporting Information S2 Fig). In addition, in Table 4, except for AGR, the 11 germination indexes were all significantly correlated with the PhytLa content of rice seeds, implying that the composite structure of phytolith and La(III) could affect the physiological and biochemical changes of germinating seeds in rice seed germination.

**Table 3:** Correlation analysis of the vigour index of germinated rice seeds.

	AGR	GR	GI	GE	GL	RL	SMLR	SMOR	GII	RII	FW	DW
AGR	1	-0.6333**	-0.6423**	-0.6975**	-0.6138**	-0.3734*	-0.7253**	-0.6205**	0.5474**	0.3799*	-0.4577**	-0.4598**
GR		1	0.9962**	0.7782**	0.7515**	0.6623**	0.8213**	0.8705**	-0.7621**	-0.6403**	0.6667**	0.0200
GI			1	0.7638**	0.7545**	0.6619**	0.8144**	0.8646**	-0.7649**	-0.6410**	0.6726**	0.0400
GE				1	0.7605**	0.5319**	0.8256**	0.7773**	-0.7374**	-0.5274**	0.5254**	0.1844
GL					1	0.7943**	0.7517**	0.8447**	-0.9344**	-0.7840**	0.7595**	0.1225
RL						1	0.6177**	0.8378**	-0.8638**	-0.9859**	0.9045**	0.2387*
SMLR							1	0.8783**	-0.7273**	-0.6051**	0.6158**	0.2207
SMOR								1	-0.8541**	-0.8130**	0.7859**	0.0224
GII									1	0.8583**	-0.8405**	-0.2291
RII										1	-0.9312**	-0.2225
FW											1	0.1970
DW												1

* P < 0.05;

** P < 0.001;

n = 48.

**Table 4:** Relationship between the PhytLa content and the vigour index.

x	y	Linear regression equation	Correlation Coefficient (R)
PhytLa	AGR	y = 12.004x + 1.5678	0.1378
	GR	y = -54.196x + 96.646	0.4194*
	GI	y = -4.1734x + 6.9036	0.4392**
	GE	y = -48.786x + 92.599	0.2818*
	GL	y = -193.9x + 30.046	0.5677**
	RL	y = -898.95x + 55.611	0.6848**
	SMLR	y = -112.66x + 18.42	0.3154*
	SMOR	y = -174.19x + 21.583	0.5265**
	GII	y = 549.54x − 4.6991	0.6104**
	RII	y = 1624.4x − 0.9779	0.6946**
	FW	y = -23.635x + 4.1899	0.6819**
	DW	y = -1.4792x + 1.2872	0.4664**

* P < 0.05;

** P < 0.001;

n = 48.

**Fig 6:**
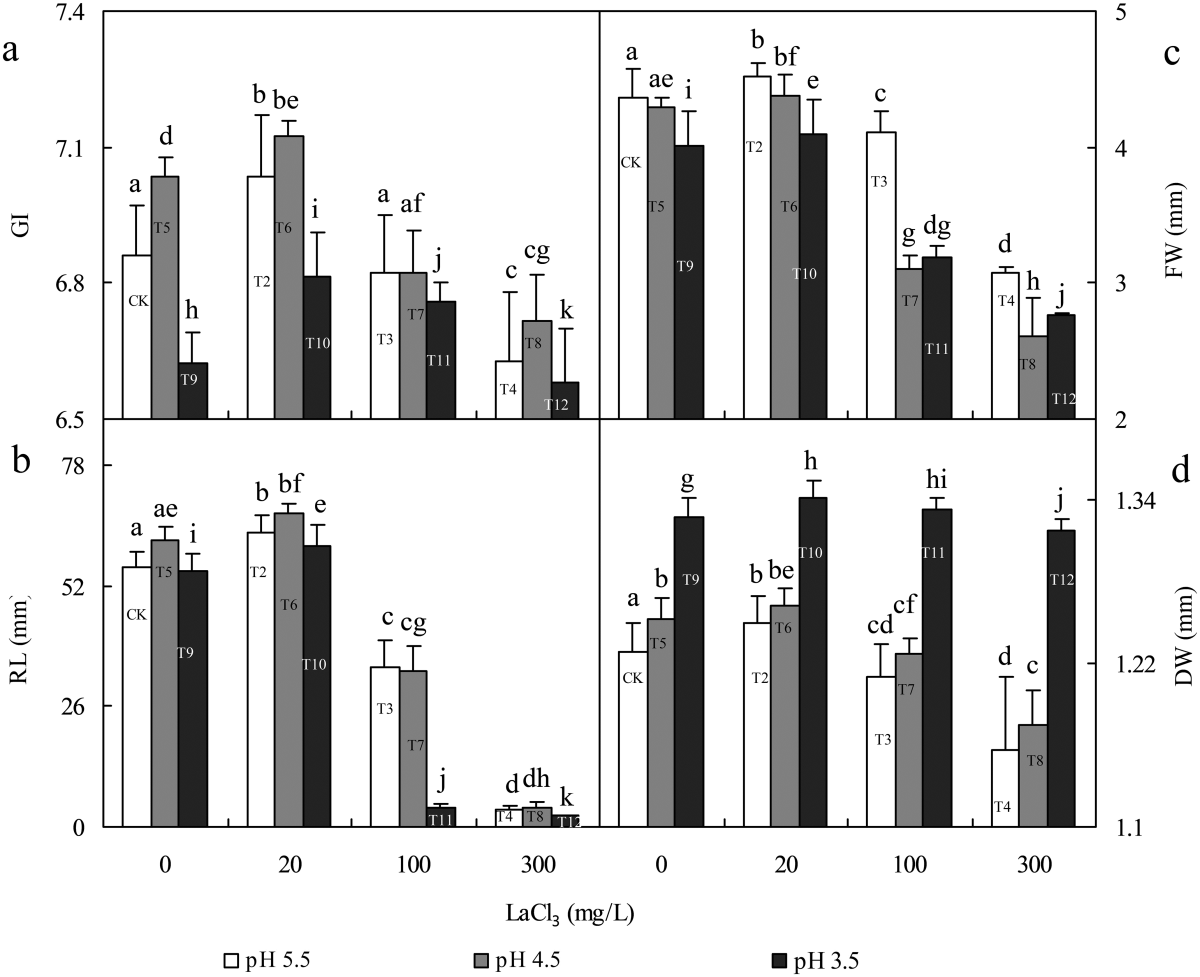
Effects of La (at LaCl_3_ concentrations of 0, 20, 100 and 300 mg/L) and acid rain (at a pH of 3.5, 4.5 or 5.5) on the seed vigor index of rice germinated seeds [GI (a), RL (b), FW (c) and DW (d)]. Error bars are standard deviations (n = 4). Different letters indicate significant differences between different treatments at P = 0.05 based on the least significant difference (LSD) test.

In the environment, La(III) and pH are both important environmental factors for regulating La(III) sequestration by phytolith in germinated rice seeds. To reveal the mutual relationship between exogenous La(III), pH and PhytLa in rice seeds and the relationship between the formation of the phytolith-La(III) complex and the physiological changes of germinated rice seeds, we compared the data on the 4 selected germination indexes (Fig 6) with those of phytolith parameters (Fig 4) and found the following rules:

First, in the single treatment with acid rain (T5, T9), compared with the CK, more energy was consumed (FW decreased) in rice seeds to relieve acid rain stress (GI increased in T5), while material accumulation increased (DW increased), but the formation of phytolith was inhibited (phytolith content decreased in T5 and T9). Compared with the CK, GI and RL in mild-acidic (pH 4.5) T5 were both promoted but were both inhibited in T9 with pH 3.5. Meanwhile, PhytLa content, PLCS and PPLCR in T5 were all promoted, and T9 showed the opposite effect. The promotional effect of mild acid (pH 4.5) on seed germination might be that rice was an acid-tolerant crop, so mild acid did not inhibit rice growth but rather stimulated rice growth and promoted the formation of PhytLa; however, severe acid had adverse effects.

Second, compared with CK, a low La(III) concentration (20 mg/L) promoted seed germination in T2 (GI and RL increased), the energy consumption in T2 decreased (FW increased), and phytolith formation (phytolith content increased), material accumulation (DW increased), PhytLa content, PLCS and PPLCR in T2 all increased significantly. In addition, compared with CK, high La(III) concentrations (100 or 300 mg/L) inhibited seed germination (GI and RL decreased) and phytolith formation (phytolith content decreased) in T3 and T4 but promoted PhytLa content significantly. Therefore, La(III) (low or high concentrations) can promote the formation of the phytolith-La(III) complex in germinated rice seeds, and the promotional effect was more significant with a higher concentration of La(III), but the formation of phytoliths and the physiological changes of rice seeds could barely affect the formation of the phytolith-La(III) complex, so phytoliths likely sequester La(III) in rice seeds due to their own structural characteristics. In order to resist La(III) stress (T3 and T4), germinated rice seeds accelerated the process of cell silicification (the phytolith content in T3 increased) and consumed more energy (FW in T3 and T4 decreased), but La(III) sequestration by phytoliths was not affected, while the PhytLa content and PLCS and PPLCR in T3 and T4 were all promoted, and more La(III) was sequestered by phytoliths in germinated rice seeds.

Third, compared with the single treatment with acid rain (T5 and T9), the GI, RL, FW and DW in the combined treatment with low La(III) and acid rain (T6 and T10) all increased, showing that the combination of low La(III) and acid rain promoted the physiological changes of germinated rice seeds, so less energy was consumed but more material was accumulated. Meanwhile, phytolith content, PhytLa content, PLCS and PPLCR all increased in T6 and T10, proving that the combination of low La(III) and acid rain not only promoted rice seed growth and the formation of phytolith but also promoted the formation of the phytolith-La(III) complex. In addition, compared with the single treatment with low La(III) (T2), the GI, RL, DW and phytolith content in T6 all increased, but FW decreased, implying that more energy was consumed in rice seeds to promote rice seed growth and development, thus promoting material accumulation and phytolith formation. However, compared with T2, the physiological changes in germinated rice seeds (GI, RL and FW all decreased) and the formation of phytoliths (phytolith content decreased) in T10 were both inhibited, but more material was accumulated (DW increased), showing that although more energy was consumed in rice seeds, the growth and development of germinated rice seeds and the formation of phytoliths were inhibited. Meanwhile, compared with T2, the PhytLa content, PLCS and PPLCR in T6 and T10 all decreased, showing that the combination of low La(III) and acid rain had inhibitory effect on the formation of the phytolith-La(III) complex. Therefore, though the combination of low La(III) and acid rain had certain promotional effects and could stimulate rice seed development and the formation of phytoliths, it also had an inhibitory effect on La(III) sequestration by phytoliths, and the inhibitory effect was more significant with lower pH values in the environment under low concentrations of La(III), so the promotion of physiological changes and phytolith accumulation in germinated rice seeds could only barely alter the inhibitory effect.

Fourth, in the combined treatment with high La(III) concentrations (100 or 300 mg/L) and acid rain (pH 4.5 or 3.5) (T7, T11, T8 and T12), compared with the single treatment with acid rain (pH 4.5 or 3.5) (T5 and T9), the GIs in T7, T8 and T12 were all inhibited, and the RL, FW and DW in T7, T11, T8, T12 were also inhibited, showing that more energy was consumed in rice seeds, less material was accumulated, and seed growth and development were inhibited. However, phytolith content, PhytLa content, PLCS and PPLCR in T7, T11, T8 and T12 all increased significantly. This inverse change trend indicated that it was difficult for rice seeds to influence PLCS through their own physiological changes but that PLCS could be influenced by the efficiency of phytolith La(III) sequestration (PhytLa content) and affect the PPLCR. In addition, compared with single treatment with high La(III) (T3 and T4), the GIs in T7, T11 and T12 all decreased, and the RL and FW in T7, T11, T8 and T12 also decreased, so the combination of high La(III) and acid rain inhibited rice seed germination, and more energy was consumed and more material was accumulated (DW in T7, T11, T8 and T12 increased), showing that the inhibitory effect of the combination of high La(III) and acid rain on rice seeds aggravated the damage of La(III) to seeds, so the number of inactive and dead cells in rice seeds might increase in turn. Meanwhile, compared with T3 and T4, except for the phytolith content in T8 and T12, the PLCS in T8 and PPLCR in T7 increased, and the phytolith content in T7 and T11, the PhytLa content in T7, T11, T8 and T12, the PLCS in T7, T11 and T12, and the PPLCR in T11, T8 and T12 all decreased, proving that the combination of high La(III) and acid rain had a significant inhibitory effect on the efficiency, production and phytolith contribution rate of La(III) sequestration by phytoliths. These results suggested that compared with the stress of a single high La(III) concentration, acid rain aggravated the inhibitory effect of high La(III) on germinated rice seeds, so La(III) damage to seeds was aggravated and La(III) sequestration by phytoliths was inhibited to some extent. Therefore, although La(III) in the environment could significantly promote phytolith La(III) sequestration, the acidic environment could weaken this process and inhibit phytolith La(III) sequestration in germinated rice seeds.

In summary, La(III) was an important environmental factor influencing the phytolith La(III) sequestration in germinated rice seeds. Compared with the single treatment with acid rain, various concentrations of La(III) [including low and high concentrations of La(III)] could improve the phytolith La(III) sequestration significantly, so the change in phytolith La(III) sequestration in germinated rice seeds was closely related to exogenous La(III), and we deduced that this relation was determined by the structural characteristics of phytoliths. Phytoliths are mainly composed of amorphous hydrated silica [55, 82]. In this chemical structure, the different numbers of OH groups on different silicons and the structures composed of these silicons and hydroxyls are unrepeatable and have very low surface charges [83]. Fraysse et al. [36] also finds that the structure of plant phytoliths has a very low surface charge, and the surface charge of amorphous silica decreases slightly as the phytolith structure becomes more complex. The chemical activity of amorphous silica is much more reactive than that of crystalline silica (SiO_2_), and this property makes it easier for phytoliths to combine with metal and heavy metal cations [83]. The structural characteristic of phytoliths makes it easier to combine with La(III) [30], so the composite structure of phytoliths and La(III) is a very stable chemical structure that will be more vulnerable to exogenous La(III). In addition, environmental pH is also an important environmental factor affecting phytolith La(III) sequestration in germinated rice seeds. Although single acid rain promoted phytolith La(III) sequestration in germinated rice seeds, the combination of La(III) and acid rain inhibited La(III) sequestration. The reason for this result might be that a lower pH value in the environment induced a decrease in pH in the inner tissues of seeds (such as embryos and endosperms), and the surface charge of the phytolith silica structure formed by silicified cells began to increase slightly [36], thus decreasing the sequestration capacity of phytoliths for La(III), and the stability of phytolith silica-La(III) structure was weakened, so the efficiency and degree of phytolith La(III) sequestration in germinated rice seeds were inhibited. Therefore, in the environment with La(III) pollutants, we should focus on the effects of exogenous La(III) on phytolith La(III) sequestration in germinated rice seeds and simultaneously take the effects of the combination of acidic environmental factors (such as acid rain) and La(III) into account.

### Implications of phytolith La sequestration in agriculture

Seed germination is the key stage of the plant regeneration prediction model, which can determine the biomass of subsequent mature crops. The biogenic Si (BSi) pool is mainly derived from plant litters and residues [84], and phytolith silica accounts for the vast bulk of the pool of BSi [85]. Crop biomass often determines the size of the BSi pool [86]. Therefore, high probability and fast seed germination can lead to higher biomass and phytolith returns in the soils of mature crops, and vice versa. In this study, a low La(III) concentration not only promoted rice seed germination but also increased the PhytLa content, PLCS and PPLCR, suggesting that a low La(III) concentration (e.g., by REE micro-fertilizer application) could increase the biomass and yield of crops in cultivated soils [13, 87], thus increasing the BSi pool through crop residue returns and finally strengthening the sequestration potential of phytoliths on La(III) in cultivated soils. The experiments conducted by Nguyen et al. [88] demonstrated that trivalent metal reduced the solubility of phytoliths by adsorbing on the surface of phytoliths. The complexation of La(III) and phytoliths makes it more difficult for La(III) to be released anew from phytoliths [30]. Therefore, in cultivated soils, the La(III) risk caused by micro-fertilizer application might be reduced along with the return of phytoliths within crop residues. However, this study demonstrated that high La(III) concentrations inhibited rice seed germination, thus reducing the biomass of cultivated fields and the yield of crops, so cultivated soils located in rare earth mineralized areas (with high REE concentrations) had smaller BSi pools than soils far from rare earth mineralized areas. Though this study proved that a high La(III) concentration improved the efficiency and production of phytolith La(III) sequestration in germinated rice seeds, the reduction of crop biomass would result in the decrease in La(III) sequestration potential by phytoliths in cultivated soils as the return of phytoliths derived from crop residues to soils decreases inevitably.

The change in soil pH caused by acid rain and other factors is a vital factor influencing La(III) accumulation in cultivated soils. Many previous studies have indicated that the La(III) concentration in soil generally increased with decreasing soil pH value [89, 90]. The effect is most clear in acidic soils as soil with low pH value promotes the accumulation potential of La(III) because the surface of soil particles is charged with OH group, and thus, dissolved REE ions can easily form complexes, such as Ln(OH)^2+^, Ln(OH)^2+^, and Ln(OH)^4-^ [91]. Thus, the solubility of REEs generally increases with decreasing soil pH values [3, 7, 92–94]. In addition, the soil pH value is also a crucial factor influencing the solubility of phytoliths [36, 95]. Different from the solubility of primary ore and secondary ore, phytoliths have higher solubilities under alkaline environments (phytoliths have the highest solubility around pH values between 9 and 10, when H_4_SiO_4_ dissociates appreciably: H_4_SiO_4_ = H_3_SiO_4_^-^ + H^+^) [95]. This higher solubility is because along with increasing pH values, deprotonation of surface silanol groups (Si-O-H) occurs, and the siloxane bonds are broken [95]. Recently, Nguyen et al. [88] showed that the order of magnitude of the Si release from rice straw phytolith silica at pH 6.5 is much greater than that at pH 3.0, which further confirms the opinion of Fraysse et al. [95]. In this study, compared with CK (pH 5.5), in the single treatment with acid rain (pH 4.5 or 3.5), the PhytLa content, PLCS and PPLCR all increased, showing that if other environmental factors are ignored, the total amount of phytolith-La(III) complex returns in acidic cultivated soils would be higher than that in standard paddy soils (pH 5.5). The adsorption of La(III) on the Si-O sites of phytoliths (e.g., the formation of Si-O-La-O-Si) [30] diminishes the water attack on deprotonated Si-O-Si bonds, strengthens the stability of phytoliths and decreases the possibility of La(III) release anew from phytoliths into soils [96]. Therefore, the PhytLa returned to soils can accumulate persistently over a long-term period, thus reducing the environmental risk of La(III) in soils.

On the other hand, in this study, compared with the single treatment with La(III), in the combined treatment with acid rain and different La(III) concentrations, the PhytLa content, PLCS and PPLCR all tended to decrease, indicating that environments with lower pHs are a potential threat to phytolith La(III) sequestration by rice in soils with La(III) pollutants. Meanwhile, compared with the CK and the single treatment with pH, the combined treatment with low La(III) and acid rain promoted rice seed germination significantly, meaning that the biomass returns in cultivated soils will increase significantly, and more phytolith returns will occur. As mentioned above, the combination of acid rain and La(III) could make the phytolith silica structure more stable, so when low La(III) concentrations and acid condition occur at the same time, more rice biomass will be returned to cultivated fields, and the contribution of phytoliths to La(III) sequestration in soil might be very significant. If other factors are ignored, after a long period of PhytLa returns to soils, the concentration of La(III) in the soil solution is likely to decrease. Therefore, in agricultural practice, if we can maintain a low concentration of La(III) in rice soil and avoid the influence of acidic environmental factors (such as acid rain), the PhytLa returns of rice to cultivated soils will play an important and positive role in reducing the environmental risk of La(III).

## Conclusion

In summary, our study demonstrated the following three findings:

1. La(III) and pH values were two important external factors influencing the phytolith La(III) sequestration in rice seed germination. pH 4.5 promoted phytolith La(III) sequestration, while pH 3.5 inhibited La(III) sequestration, and low La(III) concentrations (20 mg/L) and high La(III) concentrations (100 and 300 mg/L) both promoted it. However, compared with the single influence of La(III), the combination of La(III) and pH had inhibitory effects on phytolith La(III) sequestration.
2. The quantity and size of phytolith morphology were two important factors influencing the phytolith La(III) sequestration in rice seed germination. Different phytolith morphotypes had different effects on phytolith La(III) sequestration, and the effect of tubes was more significant.
3. Correlation analysis showed that in germinated rice seeds, the efficiency of phytolith La(III) sequestration had no correlation with the production of phytoliths but that the production of phytoliths and the efficiency of phytolith La(III) sequestration had a significant correlation with phytolith La(III) sequestration, and the efficiency of phytolith La(III) sequestration was closely related to the physiological changes of germinated rice seeds.

## Supporting information

S1 Fig Effects of La (at LaCl_3_ concentrations of 0, 20, 100 and 300 mg/L) and acid rain (at a pH of 3.5, 4.5 or 5.5) on the content of various phytolith morphologies of germinated rice seeds [Crenates (a), Elongate smooths (b) and Elongate echinates (c)]. Error bars are standard deviations (n = 4). Different letters indicate significant differences between different treatments at P = 0.05 based on the least significant difference (LSD) test.
(TIF)

S2 Fig Effects of La (at LaCl_3_ concentrations of 0, 20, 100 and 300 mg/L) and acid rain (at a pH of 3.5, 4.5 or 5.5) on the seed vigor index of germinated rice seeds [GR (a), GE (b), GL (c), GII (d), SMLR (e), SMOR (f), AGR (g) and RII (h)]. Error bars are standard deviations (n = 4). Different letters indicate significant differences between different treatments at P = 0.05 based on the least significant difference (LSD) test.
(TIF)
