# Supplementary figures and images for: Effects of lanthanum and acid rain stress on the bio-sequestration of lanthanum in phytoliths in germinated rice seeds
Source: PLoS One. 2018 May 15;13(5):e0197365. doi: 10.1371/journal.pone.0197365 (PMC5953472; doi:10.1371/journal.pone.0197365)

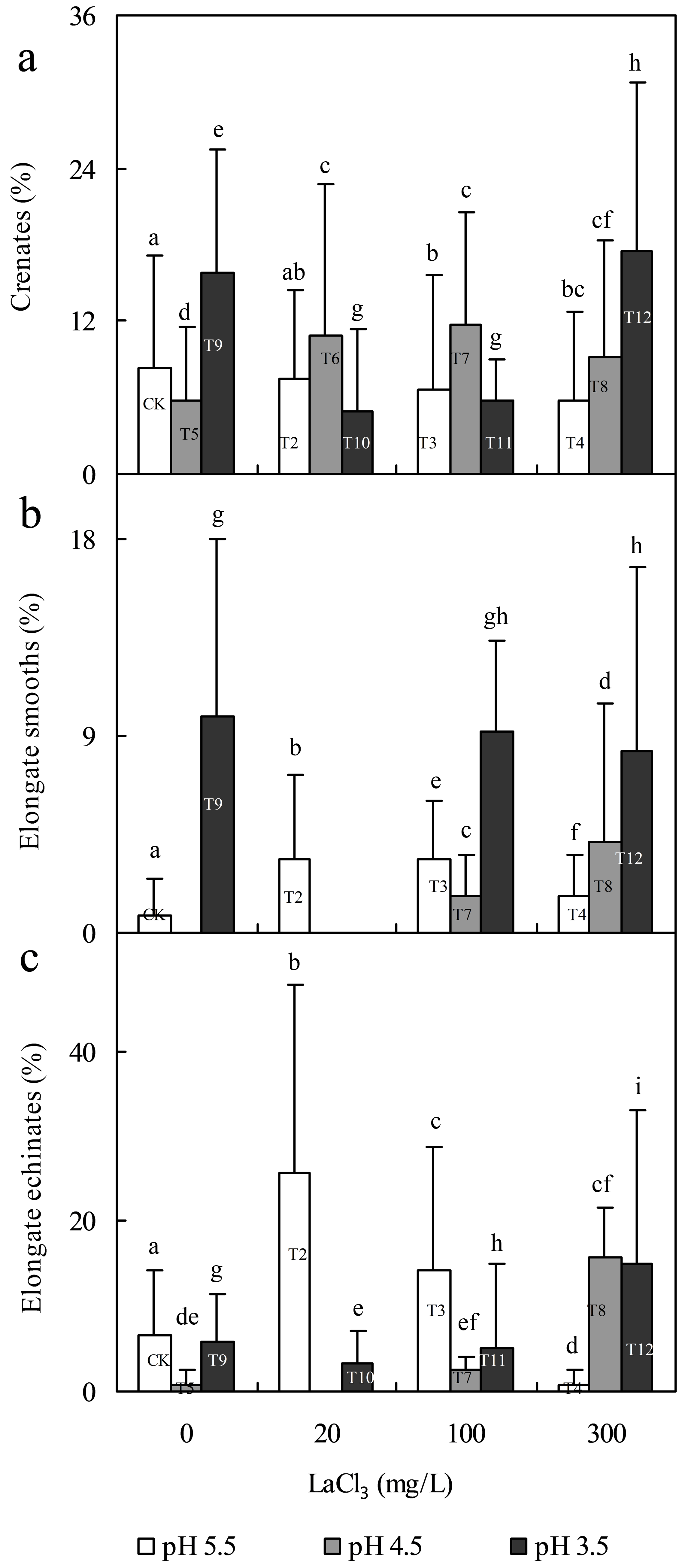

Supplement: S1 Fig — Error bars are standard deviations (n = 4). Different letters indicate significant differences between different treatments at P = 0.05 based on the least significant difference (LSD) test. (TIF) [file pone.0197365.s001.tif]

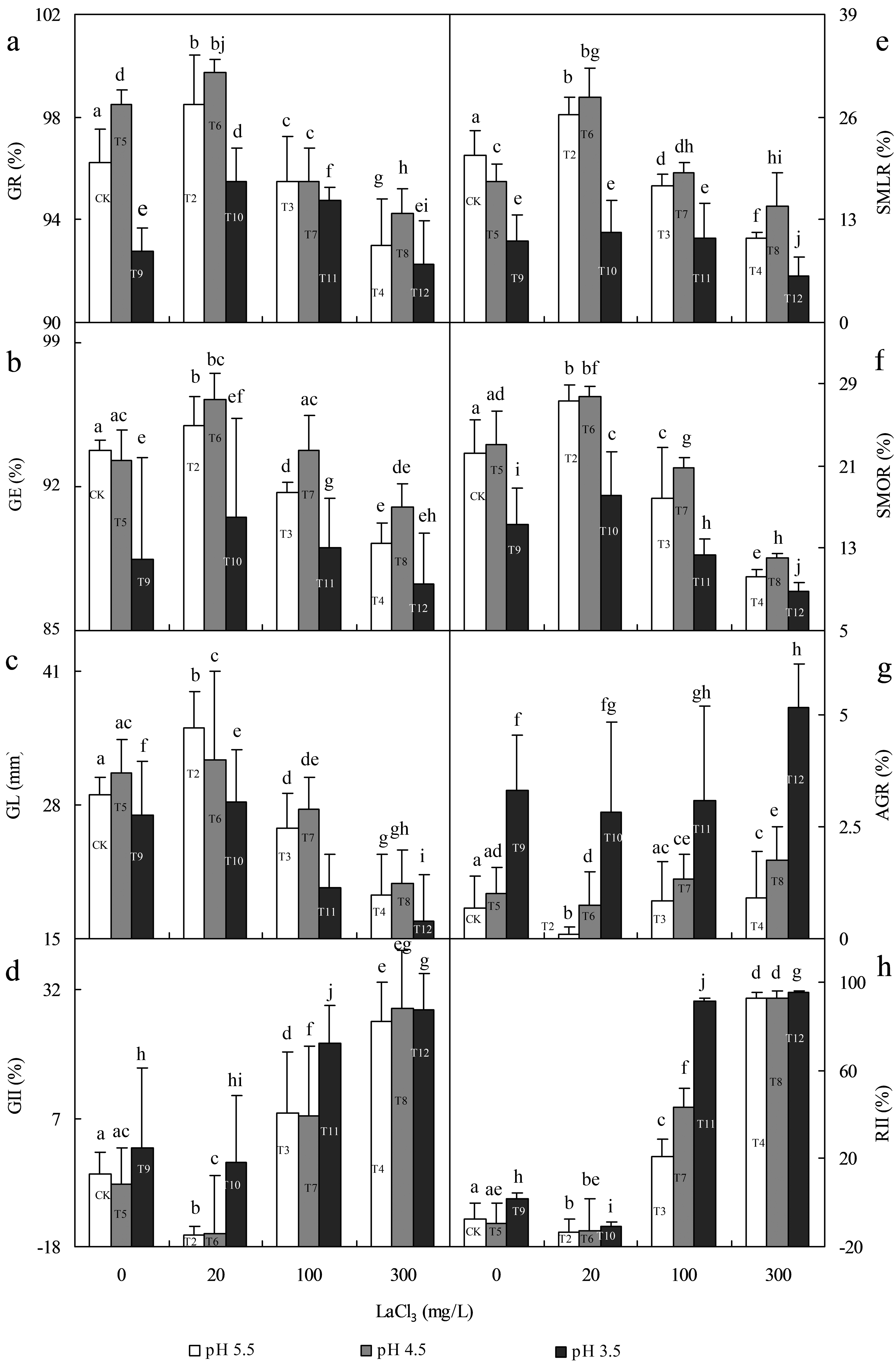

Supplement: S2 Fig — Error bars are standard deviations (n = 4). Different letters indicate significant differences between different treatments at P = 0.05 based on the least significant difference (LSD) test. (TIF) [file pone.0197365.s002.tif]
